# Small interfering RNA targeting mcl-1 enhances proteasome inhibitor-induced apoptosis in various solid malignant tumors

**DOI:** 10.1186/1471-2407-11-485

**Published:** 2011-11-14

**Authors:** Wei Zhou, Jingzi Hu, Haimei Tang, Da Wang, Xuefeng Huang, Chao He, Hongbo Zhu

**Affiliations:** 1 Department of Colorectal Surgery, Sir Run Run Shaw Hospital, School of Medicine, Zhejiang University, Hangzhou, China; 2 Key Laboratory of Biotherapy of Zhejiang province, Hangzhou, China; 3 Department of Internal Medicine, Aviation Medical Evaluation & Training center of Airforce in Hangzhou, Hangzhou, China

## Abstract

**Background:**

Targeting the ubiquitin-proteasome pathway is a promising approach for anticancer strategies. Recently, we found Bik accumulation in cancer cell lines after they were treated with bortezomib. However, recent evidence indicates that proteasome inhibitors may also induce the accumulation of anti-apoptotic Bcl-2 family members. The current study was designed to analyze the levels of several anti-apoptotic members of Bcl-2 family in different human cancer cell lines after they were treated with proteasome inhibitors.

**Methods:**

Different human cancer cell lines were treated with proteasome inhibitors. Western blot were used to investigate the expression of Mcl-1 and activation of mitochondrial apoptotic signaling. Cell viability was investigated using SRB assay, and induction of apoptosis was measured using flow cytometry.

**Results:**

We found elevated Mcl-1 level in human colon cancer cell lines DLD1, LOVO, SW620, and HCT116; human ovarian cancer cell line SKOV3; and human lung cancer cell line H1299, but not in human breast cancer cell line MCF7 after they were treated with bortezomib. This dramatic Mcl-1 accumulation was also observed when cells were treated with other two proteasome inhibitors, MG132 and calpain inhibitor I (ALLN). Moreover, our results showed Mcl-1 accumulation was caused by stabilization of the protein against degradation. Reducing Mcl-1 accumulation by Mcl-1 siRNA reduced Mcl-1 accumulation and enhanced proteasome inhibitor-induced cell death and apoptosis, as evidenced by the increased cleavage of caspase-9, caspase-3, and poly (ADP-ribose) polymerase.

**Conclusions:**

Our results showed that it was not only Bik but also Mcl-1 accumulation during the treatment of proteasome inhibitors, and combining proteasome inhibitors with Mcl-1 siRNA would enhance the ultimate anticancer effect suggesting this combination might be a more effective strategy for cancer therapy.

## Background

Proteasome inhibitors represent a new class of agents for cancer therapeutics [1,2]. The 26S proteasome is a 2, 000-kDa multimeric cylindrical complex comprising a 20S catalytic core and a 19S regulatory subunit [3]. This structure is a promising target for cancer therapy because it regulates the crucial process of proteasome-mediated protein degradation, which involves many proteins such as cyclins, caspases, Bcl-2 and the nuclear factor of κB (NF-κB) [2,4]. Inhibiting proteasome activity leads to the accumulation of these proteins, resulting in cell cycle arrest and apoptosis. Bortezomib, a specific and selective inhibitor of 26S proteasome, was approved for initial treatment of patients with Multiple Myeloma by the US Food and Drug Administration in 2008. Proteasome inhibitor-based combination therapies suggest that proteasome inhibitors could enhance chemosensitivity or reverse radiotherapy/chemotherapy resistance [5].

A growing body of evidence indicates that the intrinsic (or mitochondrial) apoptosis pathway represents a fundamental mechanism of apoptosis triggered by proteasome inhibition [6,7]. Indeed, the Bcl-2 family proteins, key activators of mitochondrial apoptosis, play a fundamental role in mediating proteasome inhibition-induced toxicity [8]. However, proteasome inhibitors not only increase the pro-apoptotic Bcl-2 proteins [9-11], but they may also lead to the accumulation of anti-apoptotic Bcl-2 proteins [12]. These proteins include the Mcl-1 anti-apoptotic protein, originally identified as an early induction gene during the differentiation of myeloid leukemia cells [13], which could block cytochrome c release from mitochondria by forming heterodimers with BH3-only proteins Bim and NOXA, or with Bak [14,15]. Thus, proteasome inhibitor-induced Mcl-1 accumulation may negatively affect their cytotoxic activity. Targeting Mcl-1 might be a strategy for enhancing the anticancer effect of proteasome inhibitors [16].

Our previous study demonstrated that proteasome inhibitors would induced a rapid Bik accumulation in various cancer cells [17]. Bik was also a member of BH3-only proteins, so the question of whether there were elevated anti-apoptotic members of Bcl-2 family existing in our system emerged inevitably. To clarify this question, we analyzed the levels of several anti-apoptotic members of Bcl-2 family in different human cancer cell lines after they were treated with proteasome inhibitors. Our results demonstrated that proteasome inhibitors induced a rapid accumulation of Mcl-1 but not others in our cell lines. The possible underlying mechanism of this accumulation might be the stabilization of proteins from degradation. We also showed that the knockdown of Mcl-1 levels by RNA interference enhanced the apoptosis induced by proteasome inhibitors. These findings suggested that treatment with proteasome inhibitors could induce Mcl-1 accumulation in various cancer cells and that combining these inhibitors with Mcl-1 siRNA might be a more effective strategy for cancer therapy.

## Methods

### Cells and cell culture

Human colon cancer cell lines DLD1, LOVO, SW620, and HCT116; human lung cancer cell lines H1299; human ovarian cancer cell line SKOV3 which were owned by our lab and human breast cancer cell line MCF7 that was purchased from ATCC, were maintained in RPMI 1640 or Dulbecco's modified Eagle's medium supplemented with 10% (v/v) heat-inactivated fetal bovine serum, 1% glutamine and 1 × antibiotics-antimycotics mixture (Invitrogen, Carlsbad, CA, USA). All cells were cultured at 37°C in a humidified incubator containing 5% CO_2_.

### Chemicals

Bortezomib was obtained from the Pharmacy of Sir Run Run Shaw Hospital and dissolved in PBS at 5 mM as a stock solution. Proteasome inhibitor MG132 and ALLN were purchased from Calbiochem (La Jolla, CA, USA) and diluted in DMSO at stock concentrations of 10 and 20 mM, respectively. Cycloheximide and DMSO were purchased from Sigma (St Louis, MO, USA). Mcl-1 siRNA and negative control siRNA were purchased from Santa Cruz Biotechnology (Santa Cruz, CA, USA). The transfection of siRNA was performed using Oligofectamine (Invitrogen, Carlsbad, CA, USA) according to the manufacturer's instructions.

### Western blot analysis

Cells were lysed in Laemmli buffer after their respective treatments. Equal amounts of lysate were separated by 10% sodium dodecyl sulfate-polyacrylamide gel electrophoresis and evaluated by Western blot analysis as described previously [18]. Rabbit anti-human caspase-9, caspase-3, Bcl-2, Bcl-XL, and Mcl-1 antibodies were purchased from Santa Cruz Biotechnology (Santa Cruz, CA, USA). Mouse anti-human PARP antibodies were purchased from BD Pharmingen (San Diego, CA, USA). Mouse anti-human β-actin was obtained from Sigma.

### Cell viability assay

The viability of the cell lines was determined by a sulforhodamine B colorimetric assay, as previously described [19]. Briefly, after fixation of adherent cells with trichloroacetic acid in a 96-well microplate, the protein was stained with sulforhodamine B, and the absorbance was determined at 570 nm to reflect the number of stained cells representing cell viability. The percentage of viable cells was determined relative to the cell viability of the PBS control, which was arbitrarily set as 1. Each experiment was performed in quadruplicate and repeated at least three times.

### Protein stability assay

To determine protein stability, we treated cells with DMSO, MG132, bortezomib, or ALLN for up to 6 h and then added cycloheximide (25 μg/ml) to block protein synthesis [20]. Collected protein samples were subjected to Western blot analysis using anti-Mcl-1 antibody. Band densities were qualified using Optimas software (Media Cybernetics, Silver Spring, MD, USA), and the mean half-life of Mcl-1 was calculated.

### Flow cytometry assay

Apoptosis was detected using an FITC Annexin-V Apoptosis Detection Kit (BD Pharmingen, San Diego, CA, USA) according to the manufacturer's instructions. The cells were digested with 0.25% trypsin, washed with cold phosphate-buffered saline (PBS) twice, and resuspended in binding buffer (1 × 10^6 ^cells/ml). Then 100 μl of the cell suspension (1 × 10^5 ^cells) was incubated with 5 μl of Annexin-V FITC and 5 μl of propidium iodide (PI) for 15 min at room temperature in the dark. The population of apoptosis cells was analyzed by flow cytometry (BD FACSCalibur, Becton Dickinson, San Jose, CA, USA).

### Statistical analysis

The data were expressed as mean ± SD. Differences among the treatment groups were assessed via ANOVA using statistical software (Statsoft, Tulsa, OK). A *P*-value of ≤ 0.05 was considered significant. Survival was assessed using the Kaplan-Meier method.

## Results

### Rapid accumulation of Mcl-1 induced by proteasome inhibitors in various cancer cells

**Figure 1:**
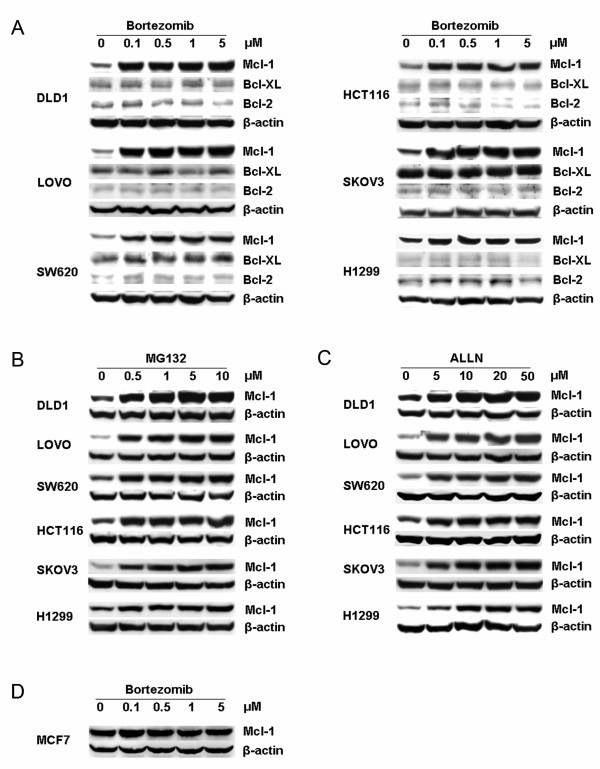
**Proteasome inhibitors induced Mcl-1 accumulation in various cancer cells**. (A) Mcl-1 but not Bcl-XL or Bcl-2 accumulation was induced by bortezomib. Western blot assay was performed after human colon carcinoma cell lines (DLD1, LOVO, SW620 and HCT116), human ovarian cancer cell SKOV3 and human lung cancer cell H1299 treated with 0.1-5 μM bortezomib; (B) Human breast cancer cell MCF7 was treated with 0.1-5 μM bortezomib. Different cell lines were treated with 0.5-10.0 μM MG132 (C) or 5-50 μM ALLN (D) for 6 h. Data represent one of two independent experiments with similar results.

In this study, we evaluated the effect of proteasome inhibitors on Mcl-1 protein expression. Firstly, Cell lines which were owned by our lab including human colon cancer cell lines DLD1, LOVO, SW620, HCT116, human ovarian cancer cell line SKOV3 and human lung cancer cell line H1299 were treated with different concentrations of bortezomib (0.1-5.0 μM) for 6 h. Proteins were then collected and subjected to Western blot assay. The results showed that Mcl-1 expression was rapidly and dramatically upregulated by bortezomib in all six cell lines (Figure 1A), even at 0.1 μM. However, bortezomib did not substantially alter the expression levels of Bcl-2 and Bcl-XL (Figure 1A). We observed similar results about Mcl-1 when we used two other proteasome inhibitors, MG132 (Figure 1B) and calpain inhibitor I (ALLN) (Figure 1C). To evaluate whether the bortezomib could also induce Mcl-1 accumulation in other cancer cells besides the cell lines used as above, we chose the human breast cancer MCF7 cell line which was purchased from ATCC. However, Mcl-1 accumulation was not observed in this cell line (Figure 1D).

**Figure 2:**
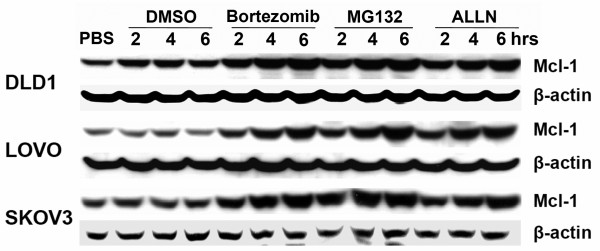
**Western blot assay for Mcl-1 expression after treatment with proteasome inhibitors at different time points**. DLD1, LOVO and SKOV3 cells were treated with 1 μM bortezomib, 5 μM MG132 or 20 μM ALLN for 2-6 h. Data represent one of two independent experiments with similar results.

To investigate whether bortezomib treatment modifies Mcl-1 accumulation in cancer cells in a time-dependent manner, DLD1, LOVO and SKOV3 cells were treated with bortezomib (1 μM) for 2-6 h. Western blot results demonstrated that in these cells, the bortezomib-induced Mcl-1 accumulation was time-dependent. Its accumulation started within 2 h after treatment and became much stronger over time. We observed similar results when we used two other proteasome inhibitors, MG132 and ALLN (Figure 2).

### Proteasome Inhibitors enhancement of Mcl-1 protein stability

**Figure 3:**
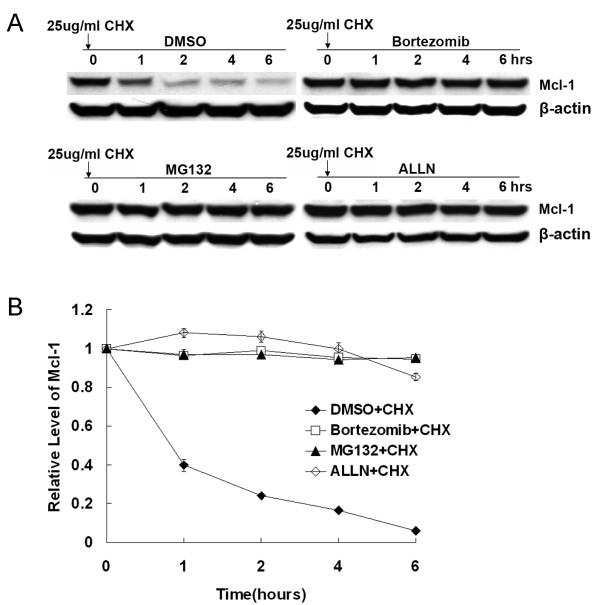
**Effect of proteasome inhibitors on Mcl-1 degradation**. (A) DLD1 cells were treated with DMSO, 1 μM bortezomib, 5 μM MG132, or 20 μM ALLN for 6 h, and then with 25 μg/ml of cycloheximide to block protein synthesis. Western blot analysis was performed. Data represent one of three independent experiments with similar results. (B) Quantitative analysis of the Western blot results shown in (A) using Optimas software. Data represent means ± SD of three assays.

Because proteasome inhibitors inhibit proteasome-mediated protein degradation, proteasome inhibitor-mediated Mcl-1 accumulation is likely to be caused by stabilization of the protein. To test this hypothesis, we treated DLD1 cells with dimethylsulfoxide (DMSO), 1 μM bortezomib, 5 μM MG132 or 20 μM ALLN for 6 h and then added cycloheximide to block protein synthesis in DLD1 cells [20]. Cells were then harvested over time and Mcl-1 levels were assessed by Western blot. Mcl-1 protein was rapidly degraded in cells treated with DMSO (Figure 3A) and had a mean half-life of less than 1 h. In contrast, in cells treated with bortezomib, MG132, or ALLN, the Mcl-1 protein level and mean half-life were stable, even after 6 h of cycloheximide treatment (Figure 3B). This result indicates that Mcl-1 degradation was blocked by treatment with proteasome Inhibitors.

### Knockdown of Mcl-1 expression by siRNA enhanced MG132-induced cancer cell death

**Figure 4:**
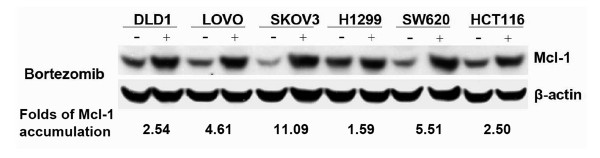
**Bortezomib-induced Mcl-1 accumulation in different cancer cell lines**. Cells were treated with 1 mM bortezomib (+) for 6 h and harvested for Western blotting. Control cells (-) received no bortezomib. Data represent one of two independent experiments with similar results. Fold of Mcl-1 accumulation represents the value of accumulated Mcl-1 normalized with its endogenous product.

Next, we studied the relationship between Mcl-1 accumulation and cells' susceptibility to proteasome inhibitors. Although the endogenous expression of Mcl-1 varied, bortezomib induced the accumulation of Mcl-1 in various cancer cells. The folds of Mcl-1 accumulation in these cell lines varied from 1.59 to 11.09 (Figure 4). Simultaneously, we also determined the susceptibility of these cells to the treatment of bortezomib. The result demonstrated that the sensitivity of cancer cells to bortezomib differed (Table 1). These results demonstrate that the cells' susceptibility to bortezomib was not obviously associated with the amount of Mcl-1 accumulation (r = 0.781, *P *= 0.066).

**Table 1:** IC50 of Different Cell Lines after exposure to bortezomib

	DLD1	LOVO	SKOV3	H1299	SW620	HCT116
bortezomib (nM)	22.5 ± 2.62	< 10	70.1 ± 14.4	25.6 ± 3.1	13.5 ± 1.9	17.7 ± 1.2

**Figure 5:**
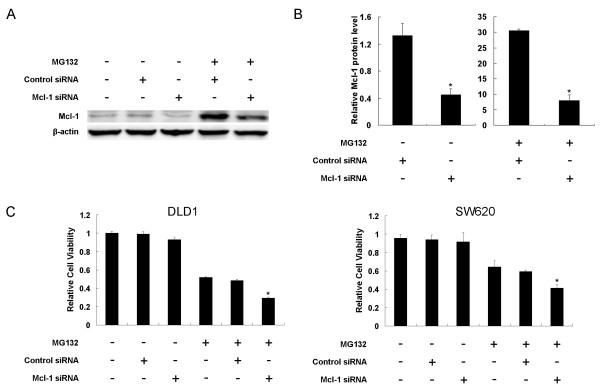
**Mcl-1 siRNA downregulated Mcl-1 protein expression and markedly sensitized DLD1 and SW620 cells to MG132-mediated cell death**. (A) Mcl-1 levels after treatment with siRNA ± MG132 in DLD1 cells. (B) Quantitative analysis of three western blot results. The value represents means ± SD. (C) MG132-mediated cell death in DLD1 and SW620 cells. Cells were pretreated with control siRNA or Mcl-1 siRNA for 24 h and then treated with 1 μM MG132 for another 24 h. DMSO was used as the mock control. Cell viability was determined in quadruplet and was normalized with cells treated with PBS alone, which was arbitrarily set as 1. The value represents means ± SD of a triplicate assay. * *P *< 0.01 compared with control siRNA+MG132.

In order to further determine the role of Mcl-1 accumulation during proteasome inhibitor treatment, we used siRNA to knock down the expression of Mcl-1 protein. For this purpose, DLD1 cells were treated with 50 nM Mcl-1 or control siRNA for 24 h; then, cells were treated with 1 μM of MG132 for another 24 h. Levels of Mcl-1 protein were determined by Western blot assay. In comparison with DMSO or control siRNA, pretreatment with Mcl-1 siRNA dramatically reduced Mcl-1 protein levels. Moreover, although treatment with MG132 still resulted in obvious Mcl-1 accumulation in cells pretreated with Mcl-1 siRNA, the level of this accumulation was dramatically lower than in cells pretreated by control siRNA (Figure 5A and 5B, *P *< 0.01). This finding also suggests that MG132-mediated Mcl-1 accumulation was extremely efficient and could occur when the Mcl-1 level was very low. Cell viability analysis showed that treatment with DMSO, control or Mcl-1 siRNA alone did not lead to cell viability loss. Treatment with MG132 for 24 h led to significant viability loss. However, in comparison with cells pretreated with control siRNA, pretreatment with Mcl-1 siRNA significantly enhanced MG132-mediated cell death (*P *< 0.01, Figure 5C). A similar result was detected in the other colon cancer SW620 cell line (*P *< 0.01, Figure 5C). Thus, a reduced level of Mcl-1 accumulation correlates with increased cell death as a result of MG132.

### Mcl-1 siRNA enhanced MG132-induced apoptotic signaling

**Figure 6:**
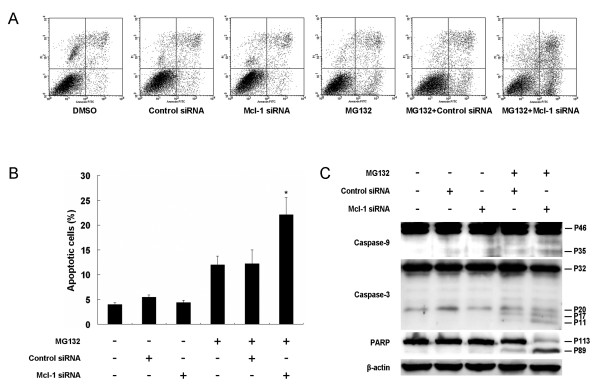
**Mcl-1 siRNA-induced sensitization to MG132-mediated apoptosis**. DLD1 cells were pretreated with control siRNA or Mcl-1 siRNA for 24 h and then treated with 1 μM MG132 for another 24 h. DMSO was used as the mock control. (A) The distribution of DLD1 cells in apoptosis measured by measured by annexin-V and propidium iodide staining using flow cytometry. Representative dot plots from one experiment of three independent experiments are shown. (B) Apoptosis percentage of DLD1 cells. The value represents means ± SD of a triplicate assay. * *P *< 0.01 compared with control siRNA+MG132. (C) Mcl-1 siRNA enhanced MG132-induced protein cleavages for caspases-9, caspase-3 and poly (ADP-ribose) polymerase (PARP). The data presented were from one of two independent experiments with similar results.

Previous studies have shown that Mcl-1 is an anti-apoptotic protein that protects tumor cells against apoptosis. Thus, it is conceivable that the knockdown of Mcl-1 contributes to MG132-induced apoptosis. To confirm this hypothesis, DLD1 cells were treated with Mcl-1 siRNA and MG132 as described above. Levels of apoptosis were determined by Annexin-V FITC/PI assay (Figure 6A). The results showed that treatment with Mcl-1 siRNA plus MG132 resulted in a significantly higher apoptosis proportion (22.07 ± 3.44%) compared to control siRNA plus MG132 (12.22 ± 2.72%, *P *< 0.01); whereas Mcl-1 (4.37 ± 0.43%) or control siRNA alone (5.48 ± 0.38%) was no more effective than the DMSO (3.94 ± 0.45%, *P *> 0.05, Figure 6B).

Furthermore, we simultaneously evaluated the cleavage of several molecular markers of mitochondrial apoptotic signaling, including caspase-9, caspase-3, and poly (ADP-ribose) polymerase (PARP), by Western blot in DLD1 cells. The results showed that in cells with a knockdown of Mcl-1, cleavage of caspase-9, caspase-3, and PARP was dramatically enhanced after treatment with MG132 compared with control siRNA-treated cells (Figure 6C). Mcl-1 downregulation by siRNA alone did not exhibit any detectable effects compared with control siRNA or DMSO.

## Discussion

Proteasomes play an essential role in degrading or processing intracellular proteins, some of which mediate cell cycle progression and apoptosis. Previous studies have shown that many types of actively proliferating malignant cells are more sensitive to proteasome blockade than non-cancerous cells [2]. Therefore, proteasome inhibitors are thought to be a novel class of anticancer drugs.

Proteasome inhibitors have a documented activity in a number of hematologic malignancies, especially in multiple myeloma and mantle cell lymphoma [21,22]. However, despite encouraging preclinical data, studies in solid tumors have yielded disappointing results [23-25]. Even in the treatment of multiple myeloma, the majority of patients do not respond, and resistance is common. The mechanism of proteasome inhibitor resistance is undefined.

Bcl-2 family members play a fundamental role in the regulation of apoptosis and are substrates of the proteasome. Previous studies implicated a role in the accumulation of pro-apoptotic Bcl-2 family members in proteasome inhibitor-induced apoptosis [16,17]. Moreover, proteasome inhibitors may also upregulate the expression of antiapoptotic Bcl-2 family members [16]. We and others have reported that treatment with proteasome inhibitors does not affect the expression of Bcl-2 and Bcl-XL [10,17,26]. However, Mcl-1 differs from Bcl-2 and Bcl-XL because it is a short-lived molecule that is highly-regulated by ubiquitin proteasome pathway [16,27-29]. The ubiquitination of Mcl-1 is mediated by Mule-a BH3-only E3 ubiquitin ligase [30]. This process requires the association of Mcl-1 with Mule and is controlled by Noxa through the regulation of the Mcl/USP9X interaction [30-32]. The level of Mcl-1/Mule complex would determine the sensitivity of cancer cells to apoptosis [33]. Therefore, Mcl-1 is likely an important survival molecule for regulating proteasome inhibitor-induced apoptosis.

In this study, we report a significant upregulation of Mcl-1 in lung cancer cell line H1299, the ovarian cancer cell line SKOV3, and the colon cancer cell lines DLD-1, LOVO, SW620 and HCT116 after treatment with different proteasome inhibitors. This effect is likely due to prolong half-life of Mcl-1. These results are similar with other previous studies, which showed that proteasome inhibitors upregulated Mcl-1 protein expression in melanoma and myeloma [16,26,34]. Previously, we had reported that proteasome inhibitors could induce Bik accumulation in various cancer cells [17]. Here we further reported that proteasome inhibitors could also induce Mcl-1 accumulation in these cells. Although both Bik and Mcl-1 protein were accumulated in these cells, they should play distinct role for cell survival. We had demonstrated that Bik accumulation induced by proteasome inhibitors might play a pro-apoptotic role in these cells [17]. Meanwhile, it had been reported that overexpressed Mcl-1 help malignant cells resistance to proteasome inhibitors [16]. Therefore, proteasome inhibitors-induced Mcl-1 in our cells may also interfere with its therapeutic effect [11,35].

To further explore the role of Mcl-1 after treatment with proteasome inhibitor, we used RNA interference to knockdown Mcl-1 levels in DLD1 cells. Our results demonstrated that although the absolute value of difference between control siRNA+MG132 group and Mcl-1siRNA+MG132 group is not so large, Mcl-1 siRNA significantly increased the cytotoxicity of proteasome inhibitors (*P *< 0.01). Our data were consistent with studies on other tumor types, such as melanoma, myeloma and malignant pleural mesothelioma, in which the specific downregulation of Mcl-1 has been shown to sensitize cancer cells to proteasome inhibitor-induced apoptosis [16,35,36]. These data suggested that Mcl-1 could partial prevent cells from death. Base on these, we don't think that Mcl-1 increase following proteasome inhibitors treatment is an epiphenomenon without a functional meaning. These results also provide a molecular basis for a rational combination of proteasome inhibitors with a Mcl-1 antagonist, such as siRNA, UV light, or fludarabine [12,16]. In the case of a potent cytotoxic with a restrictive side-effect profile [37], such as bortezomib, this combination strategy may also be effective using lower drug concentrations to avoid or minimize toxicities. Previous reports have shown that the knockdown of Mcl-1 significantly induced spontaneous apoptosis by its own [38,39]. However, we did not find obvious cell death or apoptosis after the specific downregulation of Mcl-1 in DLD1 cells, suggesting that merely losing Mcl-1 expression may not be enough to induce apoptosis. The explanation for the differential effects of Mcl-1 knockdown on the survival of different cells is not entirely clear but might reflect different expression levels of other Bcl-2 family proteins related to Mcl-1 [14].

## Conclusions

Our data showed that proteasome inhibitors induced not only Bik but also Mcl-1 accumulation in several cancer cell lines, particularly human colon cancer cell lines, and that this accumulation was mainly due to the stabilization of the Mcl-1 protein by proteasome inhibitors. The downregulation of Mcl-1 by Mcl-1 siRNA enhanced the apoptosis induced by proteasome inhibitors. Thus, using combined treatment with proteasome inhibitors and Mcl-1 antagonists may provide an effective and safe strategy for cancer therapy.

## Competing interests

The authors declare that they have no competing interests.

## Authors' contributions

WZ performed experimental and statistical analysis and drafted the manuscript. JZH and HMT participated in flow cytometry and SRB assay. DW participated in Western blot analysis. XFH and CH participated in manuscript proofreading. HBZ conceived the design, provided financial support, participated Western blot analysis and revised the manuscript. All authors read and approved the final manuscript.

## Pre-publication history

The pre-publication history for this paper can be accessed here:

http://www.biomedcentral.com/1471-2407/11/485/prepub
